# MiR130b-Regulation of *PPARγ* Coactivator- 1α Suppresses Fat Metabolism in Goat Mammary Epithelial Cells

**DOI:** 10.1371/journal.pone.0142809

**Published:** 2015-11-18

**Authors:** Zhi Chen, Jun Luo, LiuAn Ma, Hui Wang, WenTing Cao, HuiFei Xu, JiangJiang Zhu, YuTing Sun, Jun Li, DaWei Yao, Kang Kang, Deming Gou

**Affiliations:** 1 Shaanxi Key Laboratory of Molecular Biology for Agriculture, College of Animal Science and Technology, Northwest A&F University, Yangling, Shaanxi, China; 2 College of Life Sciences, Shenzhen University, Shenzhen, Guangzhou, China; 3 College of Medicine, Shenzhen University, Shenzhen, Guangdong, China; Academia Sinica, TAIWAN

## Abstract

Fat metabolism is a complicated process regulated by a series of factors. microRNAs (miRNAs) are a class of negative regulator of proteins and play crucial roles in many biological processes; including fat metabolism. Although there have been some researches indicating that miRNAs could influence the milk fat metabolism through targeting some factors, little is known about the effect of miRNAs on goat milk fat metabolism. Here we utilized an improved miRNA detection assay, S-Poly-(T), to profile the expression of miRNAs in the goat mammary gland in different periods, and found that miR-130b was abundantly and differentially expressed in goat mammary gland. Additionally, overexpressing miR-130b impaired adipogenesis while inhibiting miR-130b enhanced adipogenesis in goat mammary epithelial cells. Utilizing 3’-UTR assay and Western Blot analusis, the protein peroxisome proliferator-activated receptor coactivator-1α (PGC1α), a major regulator of fat metabolism, was demonstrated to be a potential target of miR-130b. Interestingly, miR-130b potently repressed PGC1α expression by targeting both the PGC1α mRNA coding and 3’ untranslated regions. These findings have some insight of miR-130b in mediating adipocyte differentiation by repressing PGC1α expression and this contributes to further understanding about the functional significance of miRNAs in milk fat synthesis.

## Introduction

Goat milk contains a large number of short chain fatty acids and unsaturated fatty acids, which can be used to prevent human nutrition malabsorption syndrome, small bowel dysfunction, coronary heart disease and neonatal nutritional deficiency diseases [1–3]. Thus, it is of great significance to investigate the goat milk fat metabolism [4,5]. However, in the last few decades there were rare reports about the molecular mechanism underlying this process, for which researches mainly focus on the analysis of a single gene or function verification only, lacking more comprehensive study in term of molecular regulation of milk fat metabolism [6–8].

miRNAs are a group of small noncoding RNAs with 20–23 nucleotides, which are conservative in evolution and widely exist in animals, plants, fungi and viruses [9,10]. Transcribed by RNA polymeraseII, MiRNAs are shown to regulate gene expression at post-transcriptional level [11–13]. In general, miRNAs negatively regulate target gene via binding to the 3’-untranslated region (UTR) of target mRNAs and leading to mRNA degradation or translational inhibition, through the 2–7 bases in the 5'-end of miRNA which is an important target recognition site named "seed region" [14,15]. At present, thousands of miRNAs have been found in human, and they are considered to regulate the expression of close to 30% of genes by bioinformatic prediction [16–18]. Previous studies show that more than 60% of human protein-coding genes have at least one binding site of conservative miRNAs [1,19]. Growing reports indicate that miRNAs express spatiotemporal specific manner in eukaryotic organisms and play crucial role in a variety of processes such as cell apoptosis, cell proliferation, cell differentiation, fat metabolism and insulin secretion, immune regulation, emergency response, etc. Nevertheless, there are few studies about the function and mechanism of miRNAs during the process of goat milk fat metabolism [20–22].

Many methods such as stem-loop and poly (A) assay have been developed to detect and quantify miRNA expression. S-Poly (T) real assay is a recently reported method, combining the advantages of the traditional Poly real (A) and Stem—loop methods. This method greatly enhances the anchoring strength and thermal stability of the hybridization between miRNA molecules and primers in the reverse transcription reaction, for which the primers contain specific six bases and S-Poly (T) of 11 oligo (dT) [23]. Both the miRNA detection sensitivity and specificity gain large improvement utilizing the S-Poly (T) assay. In this study, we used S-Poly (T) real method to profile 789 miRNAs expression in different period of goat mammary glands. Our results show that miR-130b express abundantly in the mammary gland of goat and is one of the miRNAs with most significantly differential expression in different periods. miR-130b has been reported to be involved in diseases, such as obesity, and lipid metabolism in adipose tissue, indicating its role in the process of lactation. To this end, we investigated the effect of elevated expression of miR-130b with miRNA mimic on milk fat synthesis in goat mammary gland epithelial cells (GMEC). Our study suggests that miR-130b plays a significant role in milk fat accumulation in goats. This is the first study which demonstrates that miRNAs participate in milk fat synthesis, and provides further insights into miRNAs functions in lactating goats.

## Material and Methods

### Ethics Statement

The animal care and use were approved by the Institutional Animal Care and Use Committee in the College of Animal Science and Technology, Northwest A&F University, Yangling, China.

### Animals, sampling, and RNA extraction

Dairy goats used in the study were taken from the elite herd of Xinong Sanen Dairy Goats in the experimental farm of Northwest A&F University of China. Three healthy goats were selected at the age of three-year-old with similar body weight in early-lactation (15 days after parturition) and peak-lactation (60 days after parturition). All goats gave birth to kids in the second lactation. We deal with those goats with euthanasia via bloodletting. Mammary gland tissues, heart, liver, spleen, lung, kidney, muscle, stomach and sebum were surgically collected from goats at the same breeding environment in the two lactations. Three samples were pooled and snap-frozen immediately in liquid nitrogen. Total RNA was extracted using Trizol reagent (Invitrogen, USA) according to the manufacturer’s instructions. The quantity and quality of RNA was determined using a Nano Drop ND-1000 spectrophotometer (Nanodrop, USA) and the RNA were stored at -80°C before use.

### Cell culture and transfection

GMEC was cultured in DMEM/F12 medium (Invitrogen Corp., USA), containing 5 mg/ml insulin, 0.25 mmol/l hydrocortisone, 50 U/ml penicillin/ml streptomycin, 10 ng/ml epidermal growth factor 1 (EGF-1, Gibco), and 10% FBS at 37°C in a humidified atmosphere with 5% CO2. GMECs were fractionated and cultured according to previous report [24]. To induce lacto genesis, GMECs were cultured in a lactogenic medium for 48h prior to initial experiments to promote differentiation into a secretory cell type as reported previously [25,26]. Cells were transfected with either the scramble or miR-130b mimic (60nM) or inhibitor (60nM) (Invitrogen, USA) using Lipofectamine TM RNAiMAX (Invitrogen, USA) according to manufacturer’s instructions. The mimic is a small chemically modified single-stranded RNA molecule designed to mimic mature endogenous miRNAs after transfection into cells, while miRNA inhibitor is single-stranded RNA molecule designed to specifically bind to and inhibit endogenous miRNA molecules. The sequence of miR-130b: 5’- CAGUGCAAUGAUGAAAGGGCAU-3’, and the sequence of siRNA: sense: 5’- GCCAACACUCAGCUAAGUUTT-3’, antisense: 5’- AACUUAGCUGAGUGUUGGCTT-3’,

### Oil red O staining

Oil red O staining was performed as described previously with modification [27]. In brief, epithelial cells transfected with either scramble or miR-130b mimic or inhibitor were washed three times in phosphate buffer solution (PBS) and then fixed in 10% paraformaldehyde for 1h. After that, the cells were stained with 5% oil red O in isopropanol for 20 min, then washed with PBS and examined microscopically. Following microscopically analysis. And then add in 400 ul of isopropyl alcohol, rapid oscillation, take 100 ul of in 96-well plates (Repeat three times). Its absorbance was determined at 510 nm. The relative fat droplet content was normalized to control transfected cells.

### Cellular triglyceride content assay

GMECs were transfected with either the scramble or the miR-130b mimic or inhibitor. After 48h of incubation, cells were harvested with lysis buffer (50mmol/l Tris-HCL, pH 7.4, 150 mmol/NaCl, 1% Triton X-100). Triglyceride was measured using a serum triglyceride kit according to manufacturer’s instructions (Loogen, China) on an XD 811G Biochemistry Analyzer (Shanghai Odin Science &Technology Company, China). The values obtained were normalized to the total protein content.

### Cell Proliferation and MTT Assays

Cells were grown in 96-well plates at a density of 5 × 10^3^ cells/well in a 200μl volume. For the 3-(4,5-dimethylthiazol-2-yl)-2,5-diphenyl tetrazolium bromide (MTT) assay, 20μl of MTT solution (5 mg/ml in PBS) was added into each well and incubated at 37°C for 4 h. Then MTT solution was removed, 150μl of dimethyl sulfoxide (DMSO) was added into each well. After 10 min of shaking at room temperature to completely dissolve formazan crystals, the absorbance was detected at 570 nm using a Microplate Reader (Dynex Technologies, U.S.A.).

### Quantitative real-time PCR (RT-qPCR) and Western blot

The mature miRNAs expression level was determined using S-Poly (T) assay [23].

The primers S3 Table were used as specific reverse primer, respectively. Briefly, reverse transcription was performed as follow: a 10 μl reaction including 0.2 μg total RNA, 2.5 μl of 4×reaction buffer, 1 μl of poly A/RT enzyme mix, 1μl of 0.5 μM RT primer. The reaction was performed at 37°C for 30 min, followed by 42°C for 30 min, then 75°C for 5 min. The RT products were amplified and detected using a universal Taqman probe, a 20 μl PCR reaction contains 0.3 μl of RT products, 4 μl of 5×qPCR probe Mix, 0.5 unit of Go Taq Hot Start Polymerase(Promega, USA), 0.2 mM universal Taqman probe, and 0.5μM forward primer and universal reverse primer, respectively. The PCR reaction was performed at 95°C for 3min, followed by 40 cycles of 95°C for 10 s and 60°C for 30 s. 18S rRNA was used as internal control. For mRNA, 0.5 μg of total RNA was synthesized into cDNA using the Prime Script RT Reagent Kit (Perfect Real time, Takara, Japan). RT-qPCR assays were performed according to the manufacturer’s instructions(SYBR Premix Ex Taq II,Perfect Real Time, Takara, Japan) with the specific primers were reported previously [26,28,29]. The expression was normalized to GAPDH. All real-time reactions, including controls with no templates, were carried out on a Bio-Rad CFX96 real-time PCR detection system (Bio-Rad, USA) in triplicate. Relative expression was calculated using 2^-ΔΔCt^ method [24,30–32].

For western blot analyses, cells were collected and lysed in RIPA buffer (Solarbio, China) supplemented with PMSF (Pierce, USA). Proteins were separated by SDS-PAGE, transferred to nitrocellulose membrane (Millipore, USA) and probed with the primary antibodies polyclonal rabbit anti-FASN(Bioss,bs-1498R,USA) and monoclonal mouse anti-GAPDH(CWBIO, CW0266, China), respectively. Polyclonal goat anti-rabbit HRP-conjuated IgG (Tiangen, China) was used as secondary antibody. All antibodies were used according to the manufacturer’s instruction. Signals were detected using the chemiluminescent ECL Western blot detection system (Pierce, USA).

### Luciferase reporter assay

To generate reporter constructs for luciferase assays, about 55 bp segment containing predicted miRNA target site in the 3’UTR of PGC-1α was synthesized by company and inserted into the psiCHECK-2 vector (Promega, USA) between the *Xho I* and *Not I* sites immediately downstream of the Renilla luciferase gene. Up sequence of wild type: 5’-*ccgctcgag*CTTTGCAGAAGCAGTGTTTCTACTTGCACTAGCATGGCCTCTGACG-3’, Down sequence: 5’-GAAACGTCTTCGTCACAAAGATGAACGTGATCGTACCGGAGACTGC*cgccggcgtataaa*-3’, Up sequence of mutant type: 5’-*ccgctcgag*CTTTGCAGAAGCAGTGTTTCTACTTTTTTTAGCATGGCCTCTGACG-3’, Down sequence: 5’- GAAACGTCTTCGTCACAAAGATGAAAAAAATCGTACCGGAGACTGC *cgccggcgtataaa*-3’, All constructs were confirmed by sequencing.

GMECs were seeded in 384-well plates at a density of 50,000 cells per well one day before transfection. 0.33 g of each reporter construct were transiently transfected using the X-treme GENE HP DNA Transfection Reagent (Roche, Switzerland) according to the manufacturer’s protocol. After a 6h recovery period in medium, cells then transfected either with scramble or miR-130b mimic or inhibitor using Lipofectamine TM RNAi MAX according to the manufacturer’s protocol. At 48 h post transfection, *firefly* and *Renilla* luciferase activities were measured with the Dual-Glo luciferase assay system according to the manufacturer’s instructions (Promega, USA).

### Statistical analysis

Statistical analyses were performed with the SPSS 18.0 statistics software package. Data are presented as mean ± SD (standard deviation) or mean ± SE (standard error) of three independent experiments. Significant differences between the groups were determined using a one-way analysis of variance (ANOVA), taking *p < 0.05, **p < 0.01 as significant differences.

### GEO accession numbers

The data obtained from miRNA sequencing studies were deposited in the Gene Expression Omnibus database at NCBI (http://www.ncbi.nlm.nih.gov/geo/). The accession number for GEO is GSE74252.

## Results

### High Throughput Screening of miRNAs in goat mammary glands at Early-lactation/dry-lactation periods

**Fig 1:**
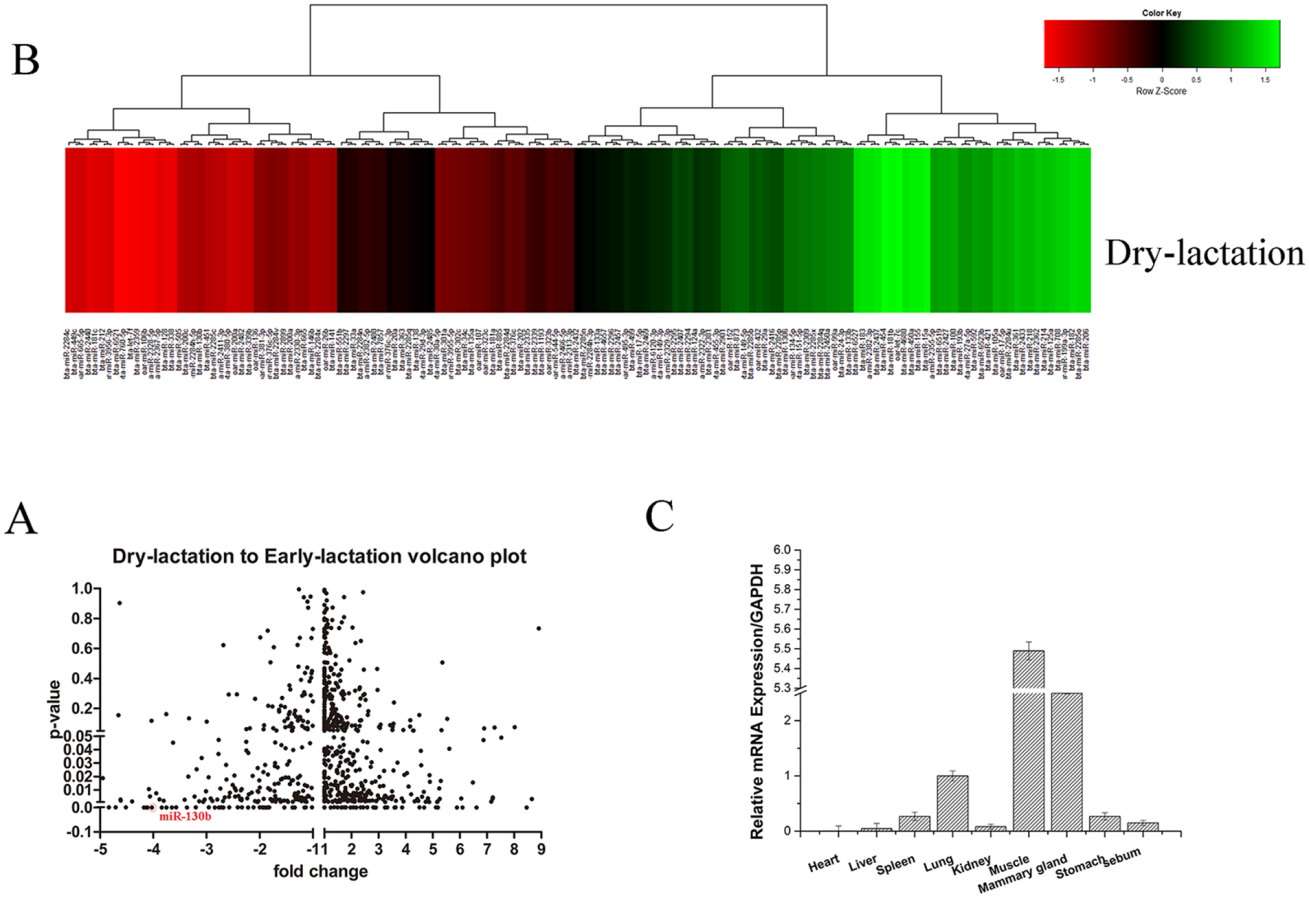
A and B: Screening for miRNAs involved in the early-lactation and dry-lactation. The samples were mixture of three goats (At the same period) in early-lactation and dry-lactation, respectively. The expression of 18s rRNA was used as a normalization control. C: miR-130b expression in various tissues of milk goats. The miR-130b expression level were detected in heart, liver, Spleen, Lung, Kidney, Muscle, Mammary gland, Stomach and Sebum. The expression of 18s rRNA was used as a normalization control. Values were presented as means ± standard errors, *, *P*<0.05; **, *P*<0.01.

Some recent reports have revealed that miRNAs are involved in the regulation of milk fat metabolism [30–32]. To investigate the connection between the miRNA regulation and this physiological process in a more comprehensive way, here we profiled the differential miRNAs expression between early-lactation and dry-lactation in goat mammary gland with S-Poly (T) real method, including 267 Capra hircus primary miRNAs and 793 Bos Taurus primary miRNAs from miRBase (http://www.mirbase.org/) and the new discovered miRNAs by Solexa sequencing. As Capra hircus and Bos taurus share high sequence homology with each other, some raw data or referenced data are overlapping. We have marked out the overlapping data, and details can be found in the additional materials (S1 Table). In the first round of screening, all the miRNAs with four -fold change and p value smaller than 0.05 were chose as candidates (Fig 1A, S1 Table). Then we took a second round of screening to confirm the expression of the candidates above and some miRNAs were excluded with the same criterion (Fig 1B, S2 Table). In the first screening, a balanced mix of different samples of goat in the same period was used. While, in the second screening, we tested samples of goat in different periods separately, and calculated the averages for each period.

Overall, we found 146 miRNAs expression changed between early-lactation and dry-lactation, with 81 up-regulation and 65 down-regulation, among which the miR-27a, miR-200a and miR-200c have been reported to involve in the milk fat metabolism process. Among these miRNAs, miR-130b is one of the most down-regulated (Fig 1A and 1B). Therefore we could focused on the miR-130b in subsequent studies. Next, we tested the miR-130b expression in different tissue of milk goats. miR-130b is majorly expressed in muscle and breast tissues (Fig 1C), supporting that miR-130b may play an important role in milk fat metabolism process.

### miR130b regulation fat metabolism in GMEC

**Fig 2:**
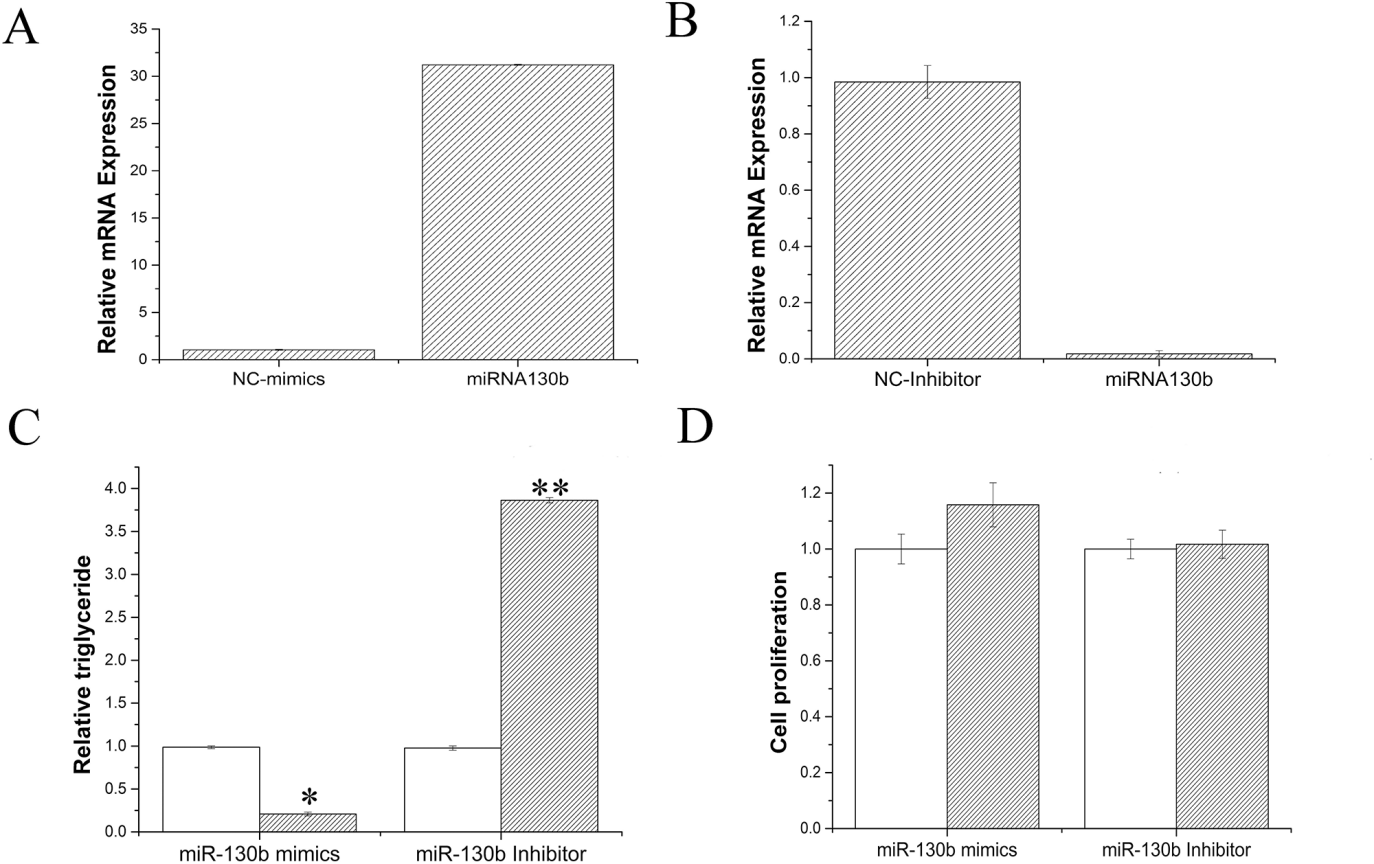
miR-130b suppresses Lipid metabolism in GMECs. A: miR-130bmimic treatment (60nM); B: miR-130b inhibitor treatment (60nM); C: Triglyceride levels in cells transfected with miR-130b mimic or inhibitor; Triglyceride levels were compared with that of control (n = 6). White bars, negative control; black bars, miR-130b mimic or inhibitor. D: Cell proliferation levels in GMECs. White bars, negative control; black bars, miR-130b mimic or inhibitor. All experiments were performed in duplicate and repeated three times. Values are presented as means ± standard errors, *, *P*<0.05; **, *P*<0.01.

To figure out the functional role of miR-130b in goat mammary gland, we used GMECs obtained from individual lactation milk goats as the research model. In the first step, we tried to investigate whether miR-130b influenced development of mammary gland, through checking the effect of miR-130b on GMEC proliferation or differentiation. Both miR-130b mimic and inhibitor were used in the research, miR-130b level was 30 times higher in miR-130b mimic transfected group than the negative control, oppositely, with 90% decrease in miR-130b inhibitor group (Fig 2A and 2B). Fig 2D showed that there were no significant difference in proliferation of GMEC between control group and experimental group (miR-130b mimic or inhibitor), indicating miR-130b didn’t impact the process of goat mammary development.

**Fig 3:**
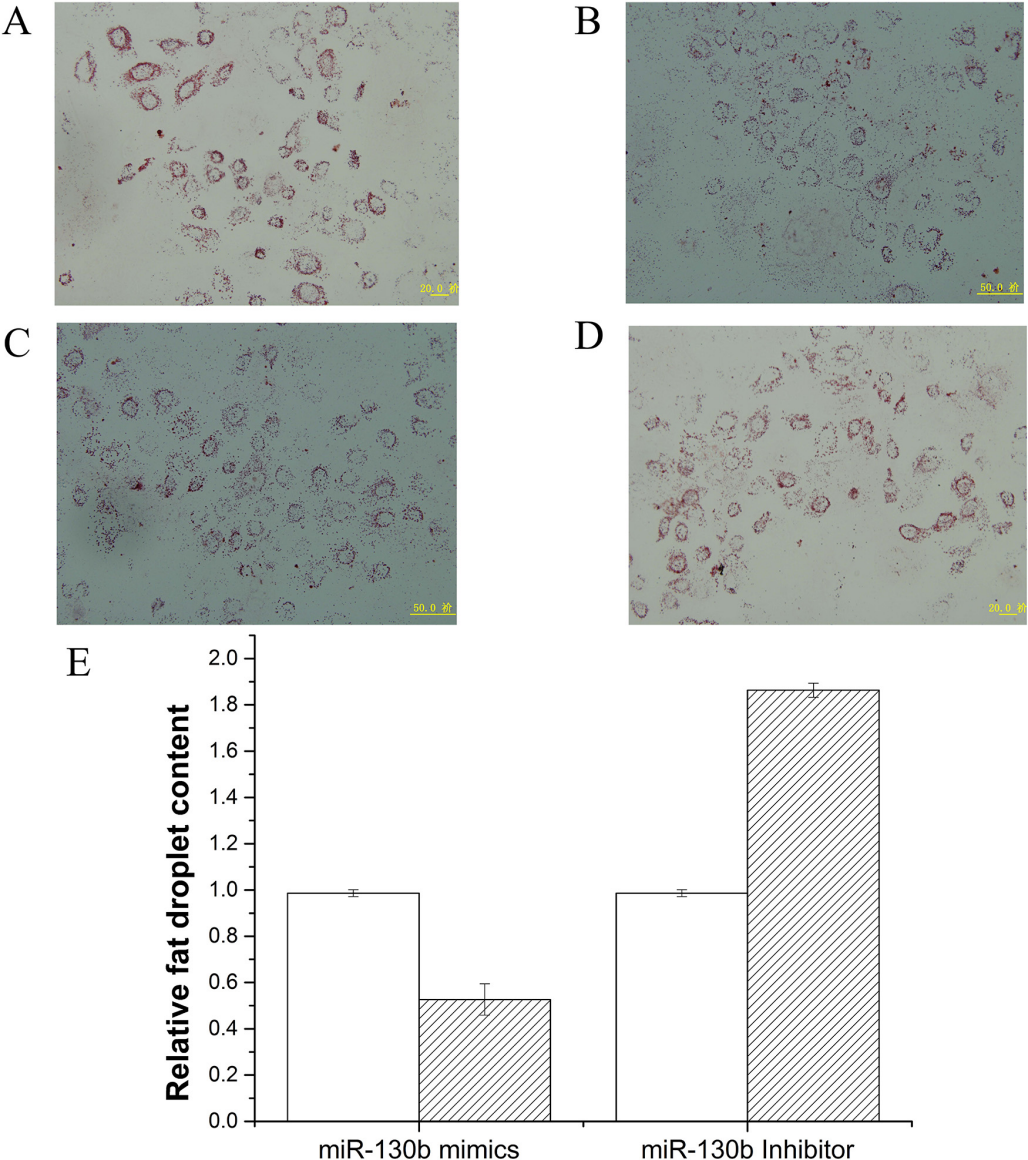
Fat droplet detection after transecting with miR-130b mimic or inhibitor in GMEC. Changes in the lipid contents of GMECs transfected with miR-130b mimic (60nM) or miR-130b inhibitor (60nM). Cells were stained by Oil Red. After examined microscopically, the oil red O was extracted with 400 μl of isopropanol and its absorbance was determined at 510 nm. The relative fat droplet content was normalized to control transfected cells. A: NC mimic treatment, B: miR-130b mimic treatment, C: NC inhibior treatment, D: miR-130b inhibior treatment. E: fat droplet content in cells. Fat droplet was compared with that of control (n = 6). White bars, negative control; black bars, miR-130b mimic or inhibitor. All experiments were performed in duplicate and repeated three times. Values are presented as means ± standard errors, *, *P*<0.05; **, *P*<0.01.

Since miR-130b didn’t involve the development of goat mammary gland, we next explored its role in milk fat metabolism. Milk fat exists as milk fat droplets which are almost completely composed of triglycerides in mammary epithelial cells [33,34]. Therefore we further measured the content of cellular triglyceride and fat droplets in GMECs, in which miR-130b was overexpressed or inhibited. As shown in Fig 2C, The triglyceride content decreased 4 times in miR-130b mimic transfected cells, compared with the negative control (P<0.05). On the other hand, triglyceride content increased significantly when miR-130b inhibited (Fig 2C). Meanwhile, we found that overexpression of miR-130b suppressed fat droplet formation, using oil red O staining (Fig 3). Moreover, fat droplet formation increased in miR-130b inhibited group (Fig 3). Our findings revealed that miR-130b plays a crucial role in milk fat metabolism and suppresses milk fat synthesis in goat mammary gland.

### MiR-130b regulates expression of genes related to fat metabolism in GMECs

**Fig 4:**
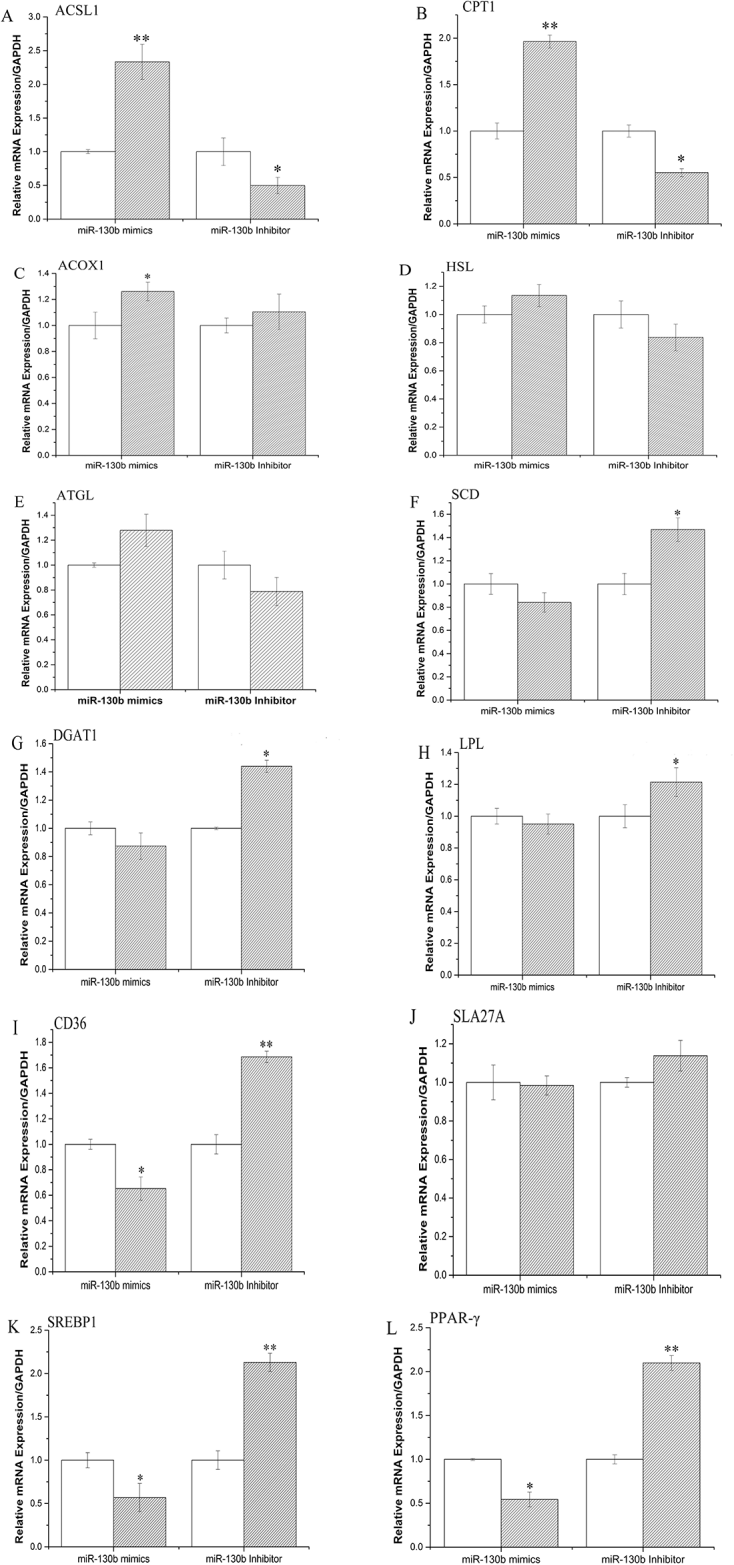
Expression of fat metabolism related genes. GMECs were transfected with miR-130b mimic or inhibitor for 48h, the mRNA expression of *ACSL*,*CPT*, *ACOX1*, *HSL*, *ATGL*, *SCD*, *DGAT1*, *LPL*, *CD36*, *SLA27A6*, *SREBP1 and PPARγ* were quantified by RT-qPCR (n = 6). White bars, negative control; black bars, miR-130b mimic or inhibitor. All experiments were performed in duplicate and repeated three times. Values are presented as means ± standard errors, *, *P*<0.05; **, *P*<0.01.

Many genes have been reported involved in the process of fat metabolism. For example, ACSL (acyl-CoA synthetase), CPT (carnitine palmitoyltransferase), ACOX1 (acyl-CoA oxidase 1) were reported to play crucial roles in β-oxidation [35], HSL (hormone-sensitive lipase) and ATGL (Adipose triglyceride lipase) are very important in Lipolysis [36]. Fatty acids outside the cell could be hydrolyzed by LPL (Lipoprotein lipase) and then transported into cells by CD36 (CD 36 molecule (thrombospondin receptor) [37]. SCD (Stearoyl-CoA desaturase (delta-9-desaturase)) and DGAT1 (Diacylglycerol acyltransferase 1) are relate to triglyceride synthesis [38]. PGC-1α and PPARγ (peroxisome proliferator-activated receptor γ) were reported to play a key role in de novo fatty acid synthesis [33,34,39]. In addition, PPARγ and SREBP1 (sterol regulatory binding protein 1) are transcriptional factors that regulate the expression of genes relevant to fatty acid and triglyceride synthesis [40]. Above we have proved the crucial role of miR-130b in the process of milk fat metabolism. Whether the expression of some fat metabolism-related genes are changed by miR-130b? To verify this, we examined the mRNA of these genes using RT-qPCR. The results showed that ectopic overexpression of miR-130b strongly up-regulated the mRNA expression of ACSL, CPT, ACOX1, HSL and ATGL (Fig 4). On the other hand, cells transfected with miR-130b inhibitor displayed marked down-regulation of several fat metabolism-related genes, such as SCD, DGAT1, LPL, CD36, SLA27A6 (Solutecarrier family 27 transporter,sub-family A,member6), SREBP1 and PPARγ (Fig 4).

### MiR-130b specifically target PGC-1α in GMEC

**Fig 5:**
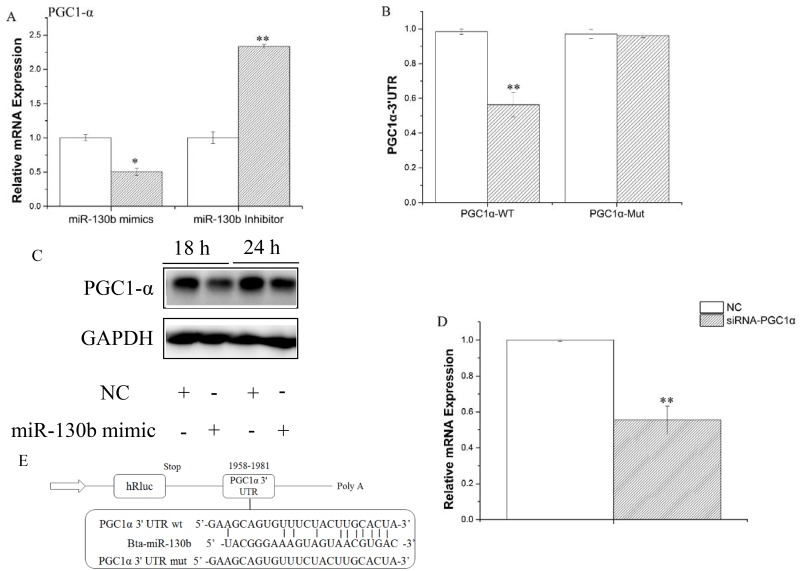
PGC-1α was identified as a target of miR-130b in GMECs. A: GMECs were transfected with miR-130b mimic or inhibitor for 48h, *PGC-1α* expression level was quantified by RT-qPCR (n = 4). White bars, negative control; black bars, miR-130b mimic or inhibitor. B and E: Target site of miR-130b in *PGC-1α* 3’UTR and the construction of the luciferase (Luc) expression vector fused with the *PGC-1α* 3’UTR. WT, Luc reporter vector with the WT *PGC-1α* 3’UTR (1958 to 1981); MU, Luc reporter vector with the mutation at miR-130b site in *PGC-1α* 3’UTR. C: Western blot analysis of *PGC-1α* expression in the miR-130b mimic and NC treatment experiments. The effect of miR-130b mimics on PGC-1α protein expression was evaluated by western blot analysis in GMECs. Total protein was harvested 24 h or 18 h post-transfection, respectively. NC, negative control. D: GMECs were transfected with siRNA of *PGC-1α* for 48h later, miR-130b expression levels was quantified by RT-qPCR (n = 6). All experiments were performed in duplicate and repeated three times. Values are presented as means ± standard errors, *, *P*<0.05; **, *P*<0.01

We used Target Scan (http://www.targetscan.org/) for prediction analysis to identify target genes of miR-130b. As goat and cattle share high sequence homology with each other, we selected a species of Bos Taurus in this process. Many genes were the potential targets of miR-130b, based on the 3’-UTR complementary prediction, such as PGC-1α. It was found in goat that the 3’-UTR of PGC-1a was targeted by miR-130b targets. We prioritized and chose PGC-1α for functional validation since is known to be a crucial regulator for multiple metabolic processes, including lipid oxidation, mitochondrial biogenesis, respiration and fibre-type switching in muscle [41]. Our results showed that mRNA of PGC-1α was down regulated by overexpression of miR-130b, and up regulated by inhibition of miR-130b (Fig 5A). Moreover, as shown in Fig 5E, goat PGC-1α indeed has a potential binding site for miR-130b in the 3’-UTR. To prove that miR-130b could directly target this site, we synthesized a 3’UTR segment of PGC-1α containing the predicted miR-130b target site (or a mutated seed site) and cloned into the psi-CHECK2 vector, for constructing a 3'-UTR reporter plasmid. Luciferase reporter assay showed that miR-130b mimic reduced the relative luciferase activity of the reporter with a wild-type 3’UTR but not the one with mutations in the seed sequences (Fig 5B and 5E). Furthermore, we detected the protein level of PGC-1α after miR-130b mimic treatment via western blotting, which was consistent with mRNA level (Fig 5C). These findings demonstrated that miR-130b directly interacted with the predicted target site of the PGC-1α mRNA and negatively regulated its expression, which at least partially explains the functional role of miR-130b during lactation. Interestingly, we also found that miR-130b could be down-regulated by knockdown of PGC-1α via PGC-1α-siRNA (Fig 5D), the significance and mechanism of which is needed further studies.

## Discussion

### miRNA expression profile at early-lactation and peak-lactation

Many previous studies have demonstrated that miRNAs plays an important role in development and lactation of breast tissue [27,42,43]. For example, Avril-Sassen has profiled and compared the expression of 102 miRNAs in different periods of lactation, of mice’s breast tissue, speculating that differentially expressed miRNAs are related to milk fat metabolic activity in mammary gland development and lactation [44]. Another group also has compared the miRNAs expression profile between pregnancy and early lactation, utilizing breast tissue of WenDeng goat in ShanDong China, and uncovered many differentially expressed miRNAs associated with breast development and lactation function. [45]. Lin Xian zi etc. found that miR—27 suppress triglycerides accumulation in mammary epithelial cells of goat through the functional study of miR-27 [32]. However, most of these studies establishing breast miRNAs expression spectrum in different period of lactation were based on sequencing and chip technology, and were rarely associated with miRNAs regulating goat milk fat metabolism and the function of mammary gland. In this study, we constructed a miRNA library which included not only 267 Capra hircus primary miRNAs and 793 Bos Taurus primary miRNAs from miRBase but also the sequencing results via Solexa sequencing, with which we screened a series of potential miRNAs involved in regulation of milk metabolism and found that miR-130b had the highest inhibitory effect on adipogenesis at least partly via targeting PGC-1α gene.

A miRNAs library with The Capra hircus (267 precursors, 436 mature) and Capra hircus (106 precursors, 153 mature) was not sufficient to explain the function and mechanism of goat mammary. To build a more comprehensive library for miRNAs screening in goat mammary, we added 793 Bos Taurus primary miRNA from miRBase and our lab’s sequencing results [31]. Herein, our library for screening contained ruminants’ miRNAs.

MiRNAs play critical roles in diverse biological processes by controlling stability and translation of mRNAs in a sequence-specific manner [46,47]. Our main purpose is to search for some known miRNAs regulating milk fat metabolism, to this end, we screened potential miRNAs with differential expression between early-lactation and peak-lactation. The Quantitative real-time PCR but not the sequencing method was closed to screen the potential miRNAs. Because if we used the sequencing method, we also needed to confirm the results via Quantitative real-time PCR finally, and some data may be lost during this process.

### MiR-130b regulates milk fat synthesis

Several reports have studied the function of miR-130b in fat metabolism. Pan et al. found that overexpressed miR-130b in HeLa-229 cells can be packaged into macrovesicles and shuttled into recipient primary cultured porcine adipocytes to reduce fat deposition by suppressing its target gene PPAR-α, involving in regulation of fat deposition[48]. Wang identified miR-130b as a potential biomarker for overweight and metabolic syndrome, and discovered a novel mechanism linking obesity and obesity-related metabolic diseases, which involved circulating miRNA-based adipose-muscle crosstalk. Their findings also broadened our awareness of the crosstalk between adipose tissue and skeletal muscle through secreted miRNA [49]. Eun Kyung Lee pointed out that miR-130 influenced PPAR-α expression and adipocyte differentiation, and highlighted the potential flame for people to understand, prevent, and manage human obesity through microRNAs [21]. However, all these researches have little exploration about the functional and mechanism role of miR-130b in lactation process.

To understand the exact function and molecular mechanism of miR-130b in mediating milk fat metabolism, we firstly carried out some function experiment, including detection of lipid contents and fat droplet formation, in the condition of miR-130b overexpression and inhibition. Interestingly, the lipid accumulation and triglyceride content were significantly increased by miR-130b mimic and repressed by miR-130b inhibitor, suggesting that miR-130b may be related to milk fat metabolism and secretion. In support of these findings, profiling of fat metabolism related gene expression in GMECs in which miR-130b was inhibited or overexpressed indicated that miR-130b could involve in lipid metabolism signaling pathways. All these results supported our hypothesis that miR-130b regulates fat metabolism in GMECs. Furthermore, we next predicted miR-130b targeted genes with bioinformatics software and selected a group of genes potentially associated with the milk fat metabolism for subsequent analysis.

### MiR-130b targeted PGC-1α 3’UTR directly

MiRNAs have long been shown to be important regulators of gene expression at the post-transcriptional level [50,51]. In plants, miRNA primarily associate with protein coding regions by extensive base pairing. In contrast, in animals, miRNA have been shown to inhibit mRNA translation and to decrease the stability of mRNA by binding to the sequences in the 3’-UTR region. PGC-1α is known to be a crucial regulator for multiple metabolic processes [52], including lipid oxidation, mitochondrial biogenesis and respiration and fibre-type switching in muscle [41].

**Fig 6:**
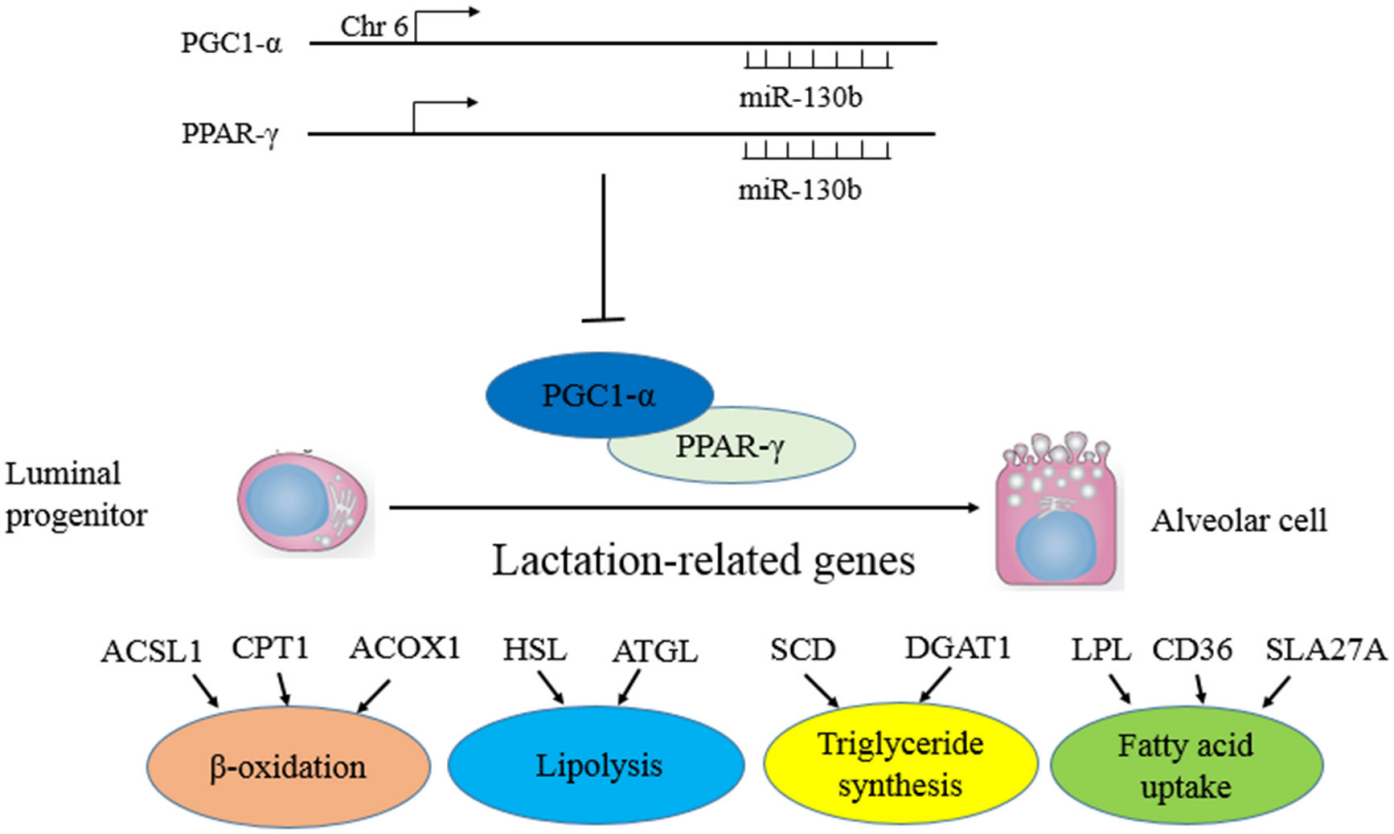
Diagram summarizing our findings miR-130b inversely target both *PGC-1α* and *PPAR-γ* in DCMECs. The down regulation of miR-130b increases *PGC-1α* and *PPAR-γ*expression levels in DCMECs.

Bonda et al demonstrated that PGC-1α lentiviral injection into the brain was tolerable and safe to be used for gene therapy in mice. These data also indicated that PGC-1α gene transfer could have therapeutic potential in AD [53]. Psilander has found that very low glycogen levels are needed to obtain a strong PGC-1a response and exercising with profoundly reduced glycogen levels might therefore be a beneficial training strategy for well-trained cyclists to promote muscle oxidative potential [41]. To confirm that PGC-1α is a real target of miR-130b, we first cloned 3′UTR of PGC-1α containing the putative miRNA regulatory element (MRE) for miR-130b into a luciferase reporter plasmid to determine whether miR-130b could have inhibitory effect. Our results showed that miR-130b significantly suppressed the luciferase activity, suggesting that miR-130b could function through the 3′UTR of PGC-1α to inhibit the reporter gene expression. In addition, we made a mutation of the potential binding site for miR-130b in the 3’UTR, and this mutation abrogated the suppressive effect ofmiR-130b on the 3′UTR of PGC-1α. Elayne Hondares has established that PPARα was an important actor in the control of BAT thermogenic activity via induction of PGC-1α and PRDM16, key players in the acquisition of the thermogenic competence of brown adipocytes [54]. Take these findings together, we hypothesize miR-130b could target and regress PGC-1α, which combine to PPARγ to regulate a large number of downstream genes, involving in milk fat metabolism. [55,56] (Fig 6).

## Conclusions

Taken together, our results have unraveled an important role of miR-130b in triglyceride accumulation and unsaturated fatty acid acceleration in GMECs for the first time. These findings will be applicable in the improvement of the quality of milk production. However, there is still much unknown about the exact mechanism, for example how the change of PGC-1α and PPAR-γ regulated by miR-130b impact lipid accumulation. This is the main works we need to take further in the future.

## Supporting Information

S1 Table Primers used first screening. Screening for miRNAs involved in the early-lactation and dry-lactation. Capra hircus primary miRNAs, Bos Taurus primary miRNAs from miRBase and overlapping data. The samples were mixture of three goats (At the same period) in early-lactation and dry-lactation, respectively. The expression of 18s rRNA was used as a normalization control.
(XLSX)

S2 Table Primers used second screening. Screening for miRNAs involved in the early-lactation and dry-lactation. Samples of goat in different periods separately, and calculated the averages for each period. The expression of 18s rRNA was used as a normalization control.
(XLSX)

S3 Table Primers used relative mRNA expression in RT-qPCR assay. Genes were clustered based on main functions relative to milk fat synthesis. GMECs were transfected with miR-130b mimic or inhibitor for 48h, the mRNA expression of *ACSL*,*CPT*, *ACOX1*, *HSL*, *ATGL*, *SCD*, *DGAT1*, *LPL*, *CD36*, *SLA27A6*, *SREBP1 and PPARγ* were quantified by RT-qPCR.
(XLSX)
